# Intracerebral Hemorrhage Induces Cardiac Dysfunction in Mice Without Primary Cardiac Disease

**DOI:** 10.3389/fneur.2018.00965

**Published:** 2018-11-20

**Authors:** Wei Li, Linlin Li, Michael Chopp, Poornima Venkat, Alex Zacharek, Zhili Chen, Julie Landschoot-Ward, Tao Yan, Jieli Chen

**Affiliations:** ^1^Department of Geriatrics, Tianjin Medical University General Hospital, Tianjin, China; ^2^Tianjin Neurological Institute, Neurology, Key Laboratory of Post-Neurotrauma Neurorepair and Regeneration in CNS, Ministry of Education and Tianjin City, Tianjin, China; ^3^Department of Neurology, Henry Ford Hospital, Detroit, MI, United States; ^4^Department of Physics, Oakland University, Rochester, NY, United States

**Keywords:** brain-heart axis, cardiac dysfunction, cardiac inflammation, intracerebral hemorrhage, oxidative stress

## Abstract

**Background:** Intracerebral hemorrhage (ICH) is a life threatening stroke subtype and a worldwide health problem. In this study, we investigate brain-heart interaction after ICH in mice and test whether ICH induces cardiac dysfunction in the absence of primary cardiac disease. We also investigate underlying mechanisms such as oxidative stress and inflammatory responses in mediating cardiac dysfunction post-ICH in mice.

**Methods:** Male, adult (3–4 m) C57BL/6J mice were subjected to sham surgery or ICH using an autologous blood injection model (*n* = 16/group). Cardiac function was evaluated at 7 and 28 days after ICH using echocardiography (*n* = 8/group per time point). Western blot and immunostaining analysis were employed to assess oxidative stress and inflammatory responses in the heart.

**Results:** Mice subjected to ICH exhibited significantly decreased cardiac contractile function measured by left ventricular ejection fraction (LVEF) and left ventricular fractional shortening (LVFS) at 7 and 28 days after ICH compared to sham-control mice (*p* < 0.05). ICH induced cardiac dysfunction was significantly worse at 28 days than at 7 days after ICH (*p* < 0.05). ICH in mice significantly increased cardiomyocyte apoptosis, inflammatory factor expression and inflammatory cell infiltration in heart tissue, and induced cardiac oxidative stress at 7 days post-ICH compared to sham-control mice. Compared to sham-control mice, ICH-mice also exhibited significantly increased (*p* < 0.05) cardiomyocyte hypertrophy and cardiac fibrosis at 28 days after ICH.

**Conclusions:** ICH induces significant and progressive cardiac dysfunction in mice. ICH increases cardiac oxidative stress and inflammatory factor expression in heart tissue which may play key roles in ICH-induced cardiac dysfunction.

## Introduction

Spontaneous, non-traumatic intracerebral hemorrhage (ICH) is a life threatening stroke subtype and a major cause of disability (1, 2). Mortality rates associated with ICH are as high as 35–52% within the first 30 days after ICH and approximately 42–65% over the first year following ICH (1). Cardiac dysfunction occurs commonly in patients and experimental animals with stroke and traumatic brain injury (TBI) (3–5). Cardiovascular complications in ICH patients are closely related with early mortality and poor outcome after ICH (6). Approximately 4% of ICH patients encounter a serious cardiac complication such as acute myocardial infarction, ventricular fibrillation, acute heart failure, and cardiac death within 2 days after stroke (7). Patients with a history of heart disease also are more susceptible to developing cardiac dysfunction after ICH, and ICH patients who developed cardiac complications typically had extended hospital stays (7). Another study reported that roughly 2% of ICH patients with or without coronary artery disease before ICH attacked developed acute myocardial infarction and these patients experienced increased risk of heart failure which were associated with increased mortality and extended hospital stays (8). Previous pre-clinical studies in mice have shown that ischemic stroke and TBI induce cardiac dysfunction characterized by significantly decreased (LVEF), cardiomyocyte hypertrophy, interstitial fibrosis, cardiac inflammatory responses, and cardiomyocyte death (5, 9, 10). However, whether ICH induces acute and chronic or progressive cardiac dysfunction in the absence of primary cardiac disease and mechanisms underlying of ICH induced cardiac dysfunction remain poorly understood.

Direct damage to brain tissue as well as products of hematoma degradation can trigger a complex cascade of pathophysiological responses including inflammatory and oxidative stress pathways which contribute to secondary brain injury after ICH (11–13). Inflammatory responses including activation of microglia and neutrophils, lead to the generation of free radicals (14). Post ICH, neutrophils are stimulated and activated, releasing large amounts of reactive oxygen species (ROS), which consume superoxide dismutase and increase of lipid peroxidation (15). In addition, oxidative stress and ROS also induces inflammation with increased expression of pro-inflammatory factors such as tumor necrosis factor (TNF), and nuclear factor-??B (16). Pro-inflammatory factors stimulate the production of ROS (16). Thus, there is a positive feedback cycle between inflammation and oxidative stress. In addition to the enhanced neuroinflammation and oxidative stress in brain after ICH, inflammation and oxidative stress are also increased in the circulation. Recent clinical studies have demonstrated that elevated serum concentration of Interleukin-6, TNF-α, matrix metalloproteinase-9 (MMP-9), and cellular fibronectin are significantly higher in ICH patients compared to healthy controls subjects and these inflammatory factors are highly associated with larger hematoma volume (17). White blood cell count increase in the peripheral is correlated with the early neurological deterioration (18). Circulating markers of oxidative stress are significantly elevated in ICH patients and are significantly and inversely correlated with long term (30 days) functional outcome (19). Thus, a growing number of clinical and preclinical studies indicate that the peripheral immune system and systemic oxidative stress are activated after ICH and may aggravate brain and systemic damage.

Human and animal studies show that immune responses and oxidative stress are involved in the pathological cascade leading to cardiac muscle dysfunction and heart failure (20, 21). Inflammatory mediators contribute to left ventricle (LV) dysfunction, LV dilation, cardiomyocyte hypertrophy, and cardiac myocyte apoptosis (22). Increased ROS contribute to atherosclerosis, restenosis, cardiac hypertrophy, cardiac fibrosis, and heart failure (23–25). Therefore, inflammation and oxidative stress may have crucial roles in mediating brain-heart interaction after ICH.

In this study, we investigated whether ICH induces cardiac dysfunction at acute and chronic phases post ICH and whether the oxidative stress and inflammation are involved in brain-heart interaction after ICH. To our knowledge, this is the first study to investigate the effects of oxidative stress and inflammation effect on “brain-heart interaction” after ICH in mice. Our results may provide potential therapeutic strategy for clinical treatment of cardiac complications after ICH.

## Materials and methods

### Animals

Adult male C57BL/6J mice (8–10 weeks old) were purchased from Vital River Laboratory Animal Technology Co., Ltd (Beijing, China). This study was conducted in accordance with the National Institutes of Health guidelines for the use of experimental animals. Experimental protocols were approved by the Tianjin Medical University General Hospital Animal Care and Use Committee. Adequate measures were taken to minimize the number of experiment animals used and to ensure minimal pain or discomfort in animals. Mice were maintained in a facility with a temperature-controlled environment on a 12 h light-dark cycle, and all animals were allowed free access to food and water.

### ICH model

To induce ICH in mice, we employed a double-injection method as described previously (26), with some modifications. Briefly, 30 μL of blood was collected within a non-heparinized capillary tube from the angular vein when the mouse was anesthetized with 5% chloral hydrate via intraperitoneal injection (7 mg/kg). Blood was quickly transferred into a 50 μL syringe with a 26 G needle (Hamilton Company). The head was fixed and held in apposition parallel to the table using a stereotactic frame. A 1 mm diameter cranial burr hole was drilled at the following coordinates relative to bregma at the injection site: X (right lateral) = 2.3 mm; Y (rostral) = 0.5 mm. A 26G needle was inserted to 3.5 mm below the surface of skull, and left in place for 5 min prior to injection. The first 5 μL was injected to generate clotting along the needle track. After an additional 5 min pause, the remaining 25 μL was injected during the subsequent 25 min at the same rate of 1 μl/min (27). After the injection was completed, the needle was left in place for 10 min to prevent reflux, before being gently removed. The burr hole was sealed using bone wax (Johnson and Johnson) and the incision closed. The body temperature was maintained using warming lamps throughout the procedure. After regaining consciousness, animals were returned to their home caged free access to food and water. Sham control mice were subjected to the same procedures as the ICH model without blood injection.

### Experimental groups

For each separate study, adult male C57BL/6 mice were randomized to two groups: (1) Sham group (total *n* = 16); (2). ICH group (total *n* = 16). Cardiac function was measured at 7 and 28 days after ICH by an investigator who was blinded to the experimental groups. One set of mice (*n* = 8/group) were sacrificed at 7 days after ICH for immunohistochemistry and Western blot assay. Another set of mice (*n* = 8/group) were sacrificed at 28 days after ICH for immunostaining analysis.

### Echocardiography measurements

Cardiac function was evaluated using transthoracic echocardiography measurements obtained using a Vevo2100 High Resolution Ultrasound System in real time (Visual Sonics Vevo 2100, Canada) with an MS-250 ultrasound scanning transducer (model C5). Mice were anesthetized with 2% isoflurane mixed with 0.5 L/min 100% O_2_ and placed in a supine position atop a heating pad maintain a steady-state sedation level throughout the procedure with 1.0–1.5% isoflurane mixed with 0.5 L/min 100% O_2_. M-mode imaging was used to obtain stable images of the parasternal long axis view. The following parameters were calculated: interventricular septum (IVS), left ventricle interior diameter (LVID), LV Volume, LVEF, and LVFS. All data were analyzed off-line at the end of the study with software resident on the ultrasound system and measured by an investigator who was blinded to the experimental groups.

### Blood pressure measurements

To test whether ICH affects blood pressure (BP), diastolic arterial pressure (DAP), mean arterial pressure (MAP) and systolic arterial pressure (SAP) were measured by tail-cuff method (CODA 8-Channel High Throughput Non-Invasive Blood Pressure system, KENT scientific) at baseline (one day before ICH) and 1, 3, and 7 days after ICH or sham surgery. The mice were habituated for 2–3 min in plastic restrainers for 7 consecutive days before experiments were performed. Body temperature was maintained at 37°C using a warming pad. Blood pressure was recorded and averaged over 15 consecutive readings.

### Western blot

Equal amounts of cell lysate from heart and plasma samples were subjected to Western blot analysis which was performed as previously described (10). Protein concentration was measured using the BCA kit (Thermos Fisher Scientific, USA). The following primary antibodies were used: interleukin-1beta (IL-1β, 1:1000, Abcam, Cambridge, MA, USA), intercellular Adhesion Molecule-1 (ICAM-1, 1:1000, R&D Systems, Minneapolis, USA), monocyte chemotactic protein-1 (MCP-1, 1:1000,Abcam, Cambridge, MA, USA), MMP-9 (1:1000, Millipore, Billerica, USA), nicotinamide adenine dinucleotide phosphate oxidase-2 (NOX-2, 1:1000, BD Bioscience, USA), transforming growth factor beta (TGF-β, 1:1000, Santa Cruz, USA), b-actin (1:10000, Abcam, Cambridge, MA, USA).

### Immunohistochemical evaluation of heart and brain

Mice were euthanized at 7 days and 28 days after ICH (*n* = 8/group in each time point). Under deep anesthesia, mouse heart and brain were harvested and fixed with 4% paraformaldehyde for 48 h and then embedded in paraffin wax. A series of sections (6 μm thick) were cut from seven coronal brain sections processed. Hematoxylin and Eosin (H&E) staining was used for hemorrhagic lesion volume calculation. Heart coronal sections (6 μm thick) were cut and Picro Sirius Red (PSR, 1:1000 dilution, Sigma, USA) staining was employed to assess cardiomyocyte cross-sectional areas (MCSA) and interstitial collagen fraction (ICF) measurement (10). For heart and brain immunostaining, antibody against CD45 (a marker for leukocyte; 1:250 dilution, Abcam,) and IBA1 (a marker for monocytes/macrophages; 1:1000 dilution, Wako, California, USA), CD206 (a M2 macrophage marker; 1:3000, Abcam, Cambridge, MA, USA), CD86 (M1 macrophage marker; 1:100; Abcam, Cambridge, MA, USA) were employed. For detecting the apoptotic cells in heart tissue, the extent of cell death was assessed and quantified by TdT-mediated Biotin-dUTP Nick End labeling (TUNEL) stain using a TUNEL kit (Millipore, Billerica, USA). Negative controls consisted of similar procedures without the addition of primary antibody.

### Immunostaining quantification

Five slides from each heart, with each slide containing four fields of view were imaged. For each mouse brain, five slides were prepared and for every slide five randomly chosen fields of view in the peri-hematoma brain region were imaged. All slides were digitized under a 20 × or 40 × objective (Olympus B × 40, Tokyo Japan) using a three-CCD color video camera (Sony DXC-970MD) interfaced with MCID image analysis system (Imaging Research, St. Catharines, Ontario, Canada). Percent of positive areas of PSR in the fields of view were calculated using image pro plus 6.0. For each field of view, cell numbers of TUNEL positive cells, CD45 positive cells, IBA1 positive cells, CD86 positive cells and CD206 positive cells were counted. A single value was obtained from averaged data and presented as percentage positive area or number of positive cells/mm^2^. Hemorrhagic lesion volume was digitally quantified using MCID image analysis system and summed from seven coronal slices at different levels. The lesion volume in cubic millimeters was calculated by multiplying the thickness by the measured areas (28). All quantification analysis was performed in a blinded fashion.

### Statistical analysis

Statistical analysis was measured by unpaired 2-tailed Student *t* test for comparison of 2 groups with use of Graph Pad Prism 5 (Graph Pad Software Inc., San Diego, CA). ^*^P < 0.05 was considered statistically significant. Data in all figures are presented as mean ± SEM.

## Results

### ICH induces progressive cardiac contractile function deficits and cardiac hypertrophy measured by echocardiography (Figure 1)

**Figure 1:**
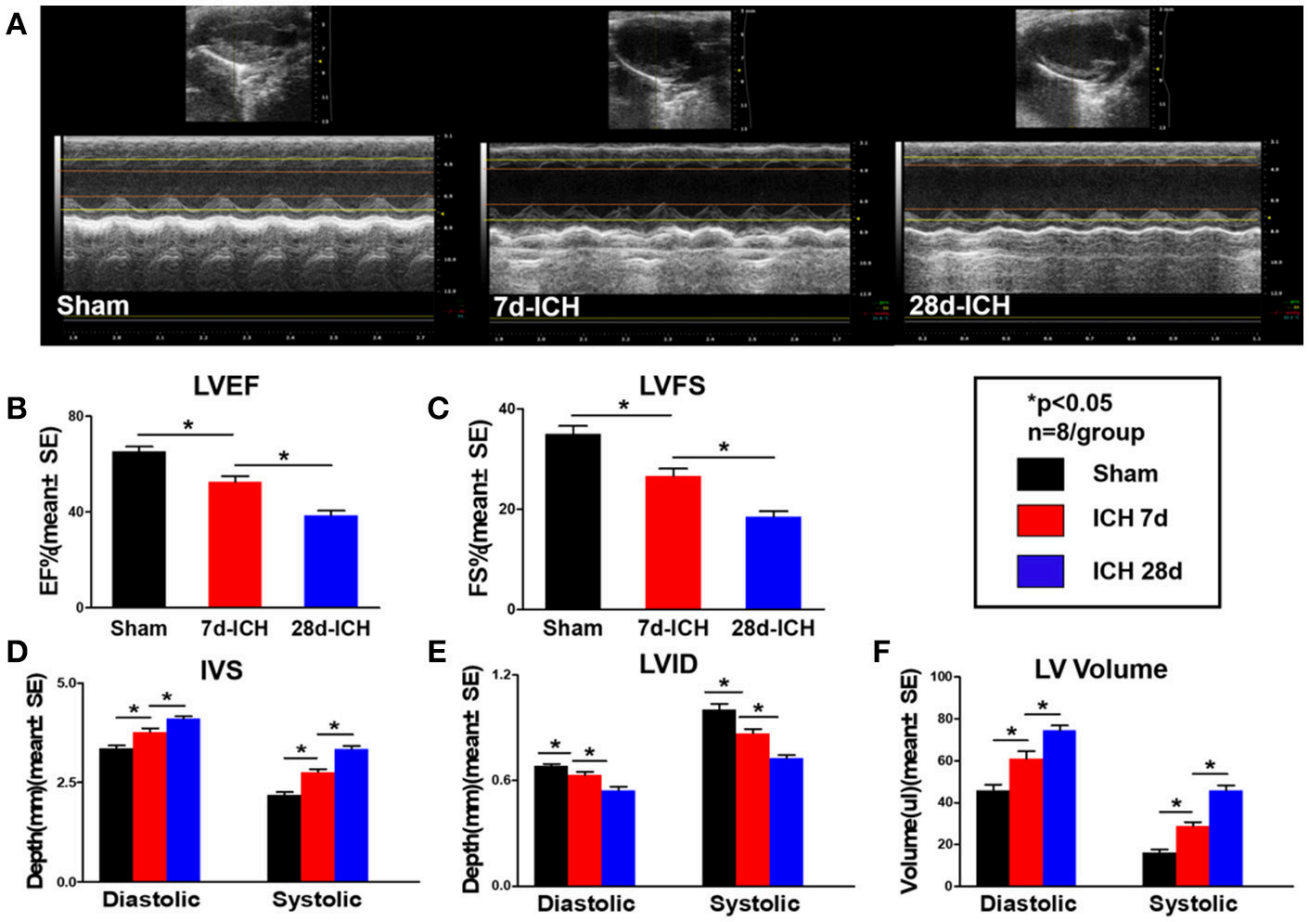
ICH induces progressive cardiac contractile functional deficits and cardiac hypertrophy measured by echocardiography. (ICH) in mice induces acute (7 days) and chronic (28 days) cardiac dysfunction. **(A)** Representative pictures of echocardiography, **(B)** left ventricular ejection fraction (LVEF), **(C)** left ventricular fractional shortening (LVFS) dimension at end diastolic and end systolic, **(D)** inter ventricular septum (IVS) dimension at end diastolic and end systolic, **(E)** left ventricle internal diameter (LVID) dimension at end diastolic and end systolic, **(F)** left ventricular volume (LV volume) dimension at end diastolic and end systolic. Bar graphs summarize the results from sham mice, ICH mice at 7 days after ICH and at 28 days after ICH. *n* = 8/group for echocardiography. Data are presented as mean ± SE; ^*^p < 0.05.

To test whether ICH induces cardiac dysfunction, echocardiography was performed on 7 days and 28 days after ICH. Figures 1A–C shows that ICH significantly decreased LVEF (B) and LVFS (C) at both 7 days and 28 days after ICH compared to sham control mice. Echocardiography results (Figures 1D–F) also show that ICH mice had significantly decreased IVS (D) and increased LVID (E), LV Volume (F) both in diastolic and systolic stage at 7 days and 28 days after ICH compared to sham control group, respectively. Compared with 7 days, the ICH mice at 28 days showed a significantly decreased LVEF (B), LVFS (C), IVS (D) and increased LVID (E), LV Volume (F), respectively (^*^p < 0.05). The data indicate that ICH induces progressive cardiac contractile function deficits and cardiac hypertrophy compared to sham control mice.

### ICH increases cardiac fibrosis and cardiomyocyte hypertrophy as well as apoptosis at 28 days after ICH. ICH does not induce any significant differences in BP at 1, 3, 7 days after ICH (Figure 2)

**Figure 2:**
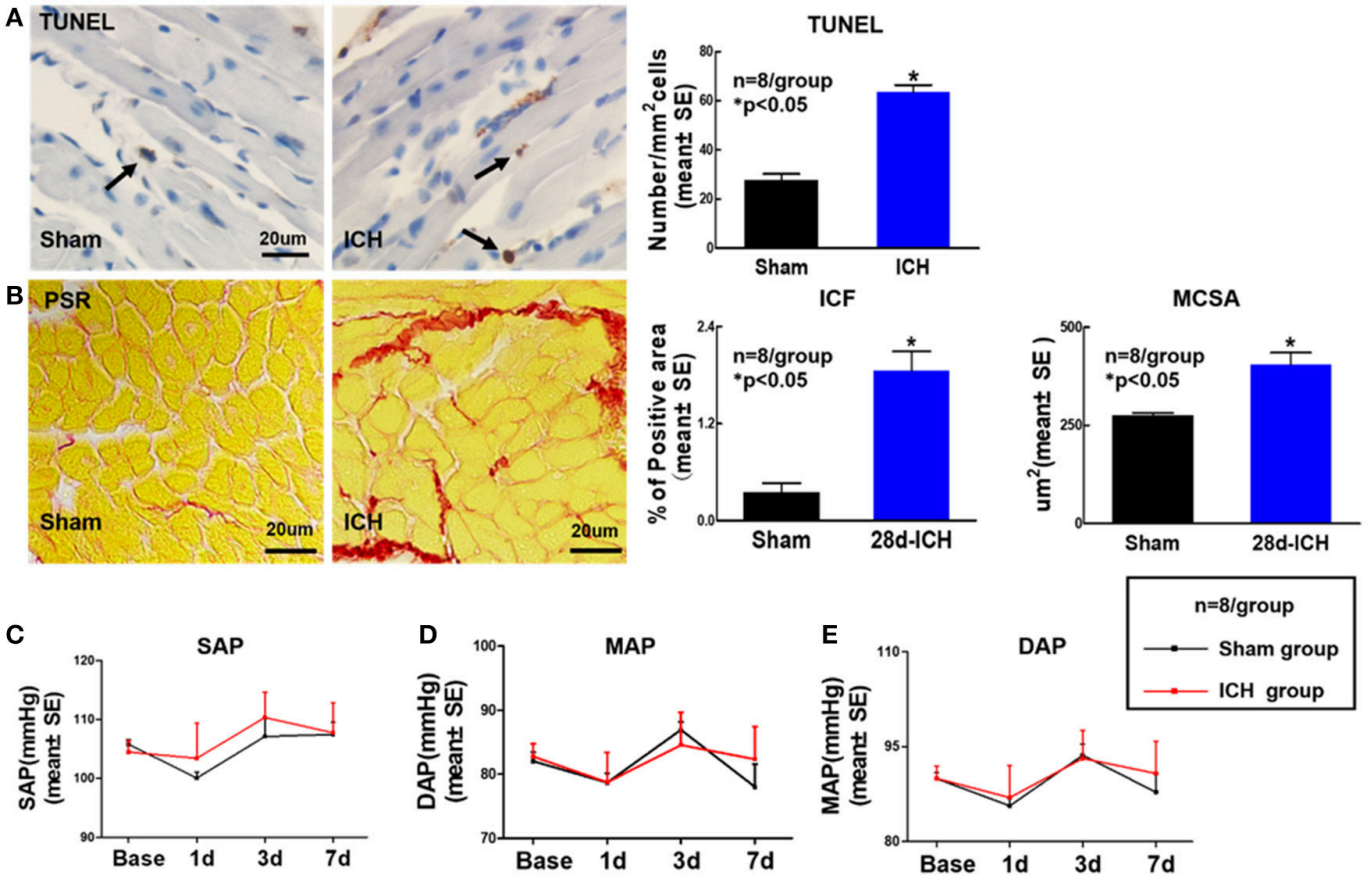
ICH increases apoptosis, cardiac fibrosis and cardiomyocyte hypertrophy at 28 days after ICH; ICH does not affect DAP, MAP, and SAP compared to sham control mice. ICH in mice induces cardiac fibrosis and cardiomyocyte hypertrophy as well as apoptosis at 28 days after ICH **(A)** Terminal deoxynucleotidyl transferase (TUNEL) staining for cardiomyocyte apoptosis and quantitative data, scale bar, 20 μm; **(B)** Picro Sirius Red (PSR) staining and quantitative data for interstitial collagen fraction (ICF) and cardiomyocyte cross-sectional areas (MCSA) measurement at 28 days after ICH, scale bar, 20 μm. **(C–E)** (DAP), mean arterial pressure (MAP) and systolic arterial pressure (SAP) measurement at 1, 3, 7 days after ICH. Data are presented as mean ± SE, ^*^p < 0.05 compared with sham control.

To determine whether ICH induces cardiac fibrosis and cardiac hypertrophy, PSR staining was performed at 28 days post ICH. Figure 2B shows that the ICH group mice exhibited significantly increased cardiac fibrosis and hypertrophy identified by increasing ICF and enlarged MCSA at 28 days after ICH compared to sham control mice. We also found that ICH significantly increases heart apoptosis quantified by TUNEL staining compared to sham control mice at 28 days after ICH (Figure 2A). The data indicate that ICH induces heart fibrosis and hypertrophy as well as apoptosis at 28 days after ICH. To test whether ICH affects BP, DAP, MAP and SAP were measured. The data (Figures 2C–E) show that ICH does not induce any significant differences in BP between sham control and ICH groups before or after surgery.

### ICH significantly increases systemic and cardiac inflammatory factor expression and oxidative stress compared to sham control mice (Figures 3, 4)

**Figure 3:**
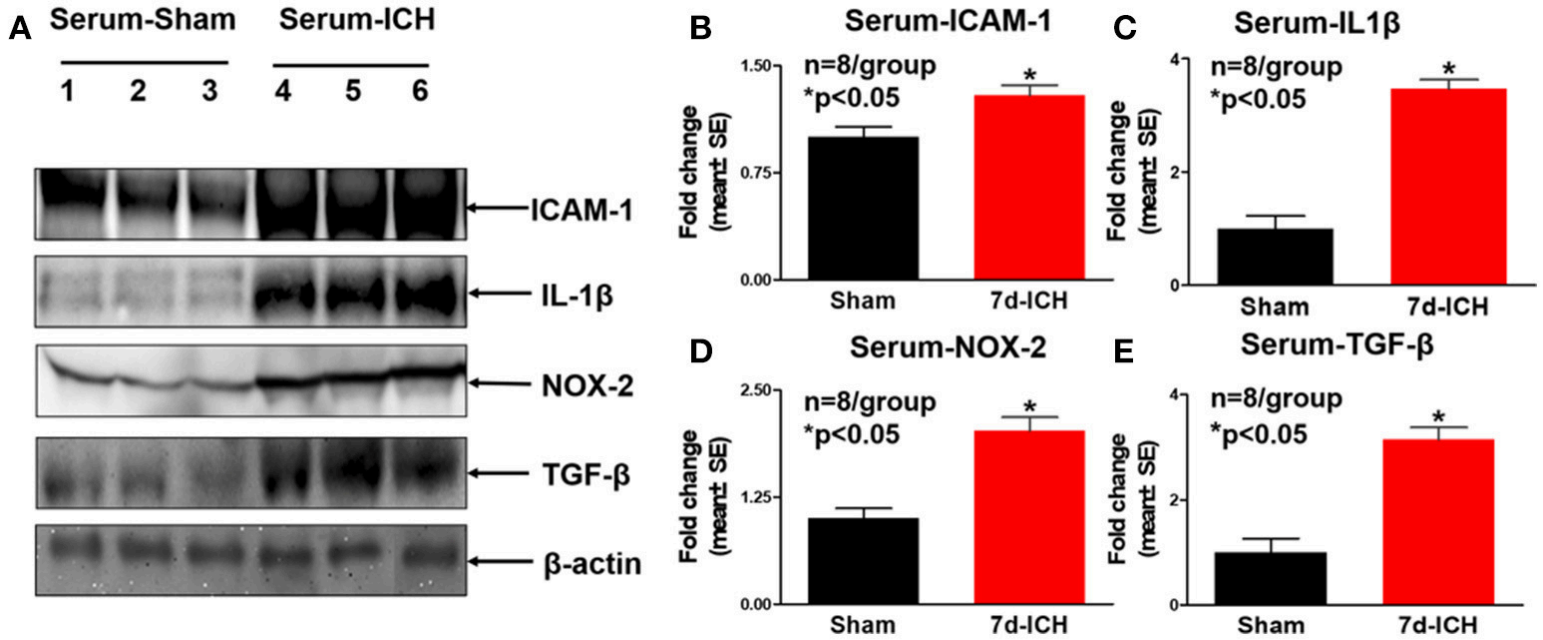
ICH significantly increases systemic inflammatory factor expression and oxidative stress compared to sham control mice. **(A)** Western blot assay shows that ICH significantly increases systemic inflammatory factor expression at 7 days after ICH such as **(B)** intercellular adhesion molecule-1 (ICAM-1), **(C)** Molecular interleukin-1beta (IL-1β) and **(E)** Transforming growth factor beta (TGF-β). ICH also significantly increases oxidative stress indicated by **(D)** NADPH oxidase-2 (NOX-2) expression in serum. Data are presented as mean ± SE, ^*^p < 0.05 compared with sham control.

**Figure 4:**
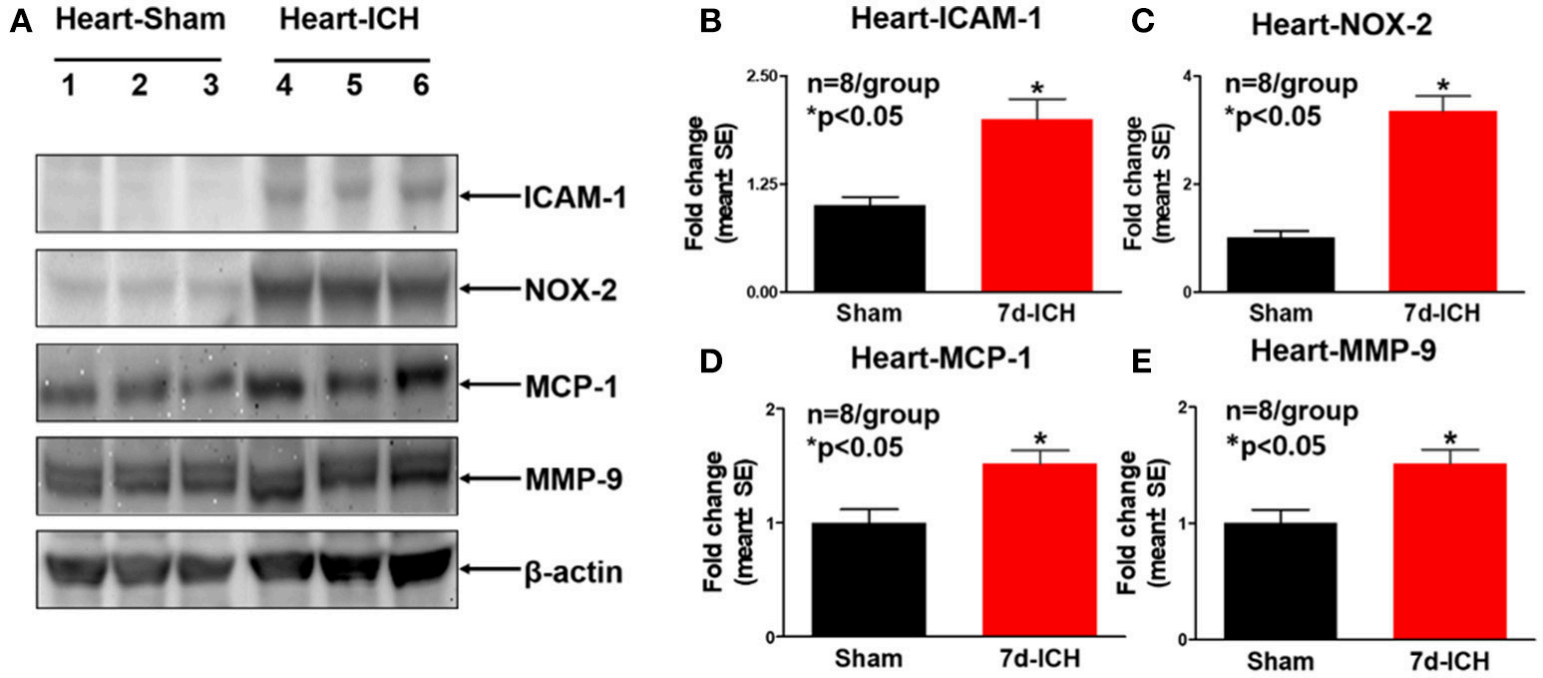
ICH significantly increases cardiac inflammatory factor expression and oxidative stress compared to sham control mice. **(A)** Western blot assay shows that ICH significantly increases cardiac inflammatory factor expression such as **(B)** intercellular adhesion molecule-1 (ICAM-1), **(C)** NOX-2, **(D)** monocyte chemotactic protein-1(MCP-1), and **(E)** matrix metalloproteinase-9 (MMP-9) in heart tissue at 7 days after ICH compared to sham control group. Data are presented as mean ± SE, ^*^p < 0.05 compared with sham control.

To investigate the mechanisms by which ICH induces cardiac dysfunction in mice, we evaluated inflammatory factor expression and oxidative stress indicator expression in serum and heart by Western blot at 7 days after ICH. Figures 3A–E show that ICH significantly increased the expression of ICAM-1(B), IL-1β (C), NOX-2 (D), and TGF-β (E) in serum compared to sham control group. Figures 4A–E show that ICH also significantly increased ICAM-1 (B), NOX-2 (C), MCP-1(D), and MMP-9 (E) expression in the heart tissue compared to sham group. The data indicate that ICH increases systemic and cardiac inflammation and oxidative stress compared to sham control mice.

### ICH significantly induces brain hemorrhage and increases inflammatory factor leukocyte and macrophage infiltration into brain at 7 days after ICH when compared to sham control mice (Figure 5)

**Figure 5:**
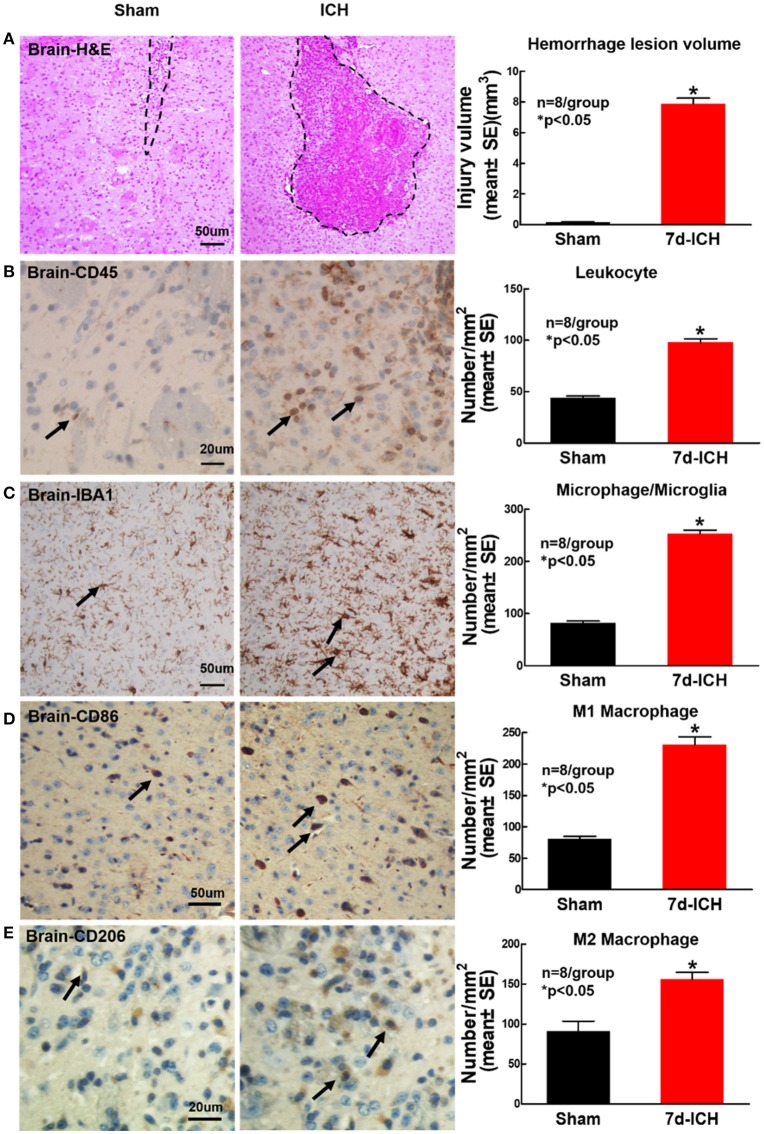
ICH model induces brain hemorrhage and increases inflammatory factor leukocyte and macrophage infiltration into brain at 7 days after ICH compared to sham control mice. **(A)** Hematoxylin and Eosin (H&E) staining images showing that ICH model in mice induces brain hemorrhage. ICH in mice significantly increases expression of **(B)** leukocyte (marker CD45, scale bar, 20 μm) and **(C)** microglia/microphage (marker IBA1, scale bar, 50 μm) in brain tissue at 7 days after ICH compared to sham control mice. ICH in mice significantly increases **(D)** M1 macrophage indicated by CD86 (scale bar, 20 μm) and **(E)** M2 macrophage indicated by CD206 (scale bar, 20 μm) in brain compared to sham control mice. Data are presented as mean ± SE, ^*^p < 0.05 compared with sham control.

To determine whether ICH induces inflammatory cell infiltration into brain at early stage after ICH, CD45, IBA1, CD206, and CD86 immunostaining was employed. Figure 5A shows that ICH significantly induces brain hemorrhage compared to sham control mice. Figures 5B,C show that ICH significantly increases inflammatory cell infiltration (CD45) and microglia/microphage expression (IBA1) as well as increases M1 (Figure 5D) and M2 (Figure 5E) macrophage expression in the peri-hematoma region of brain tissue at 7 days after ICH compared to sham control mice.

### ICH significantly increases cardiac inflammatory cell infiltration compared to sham control mice (Figure 6)

**Figure 6:**
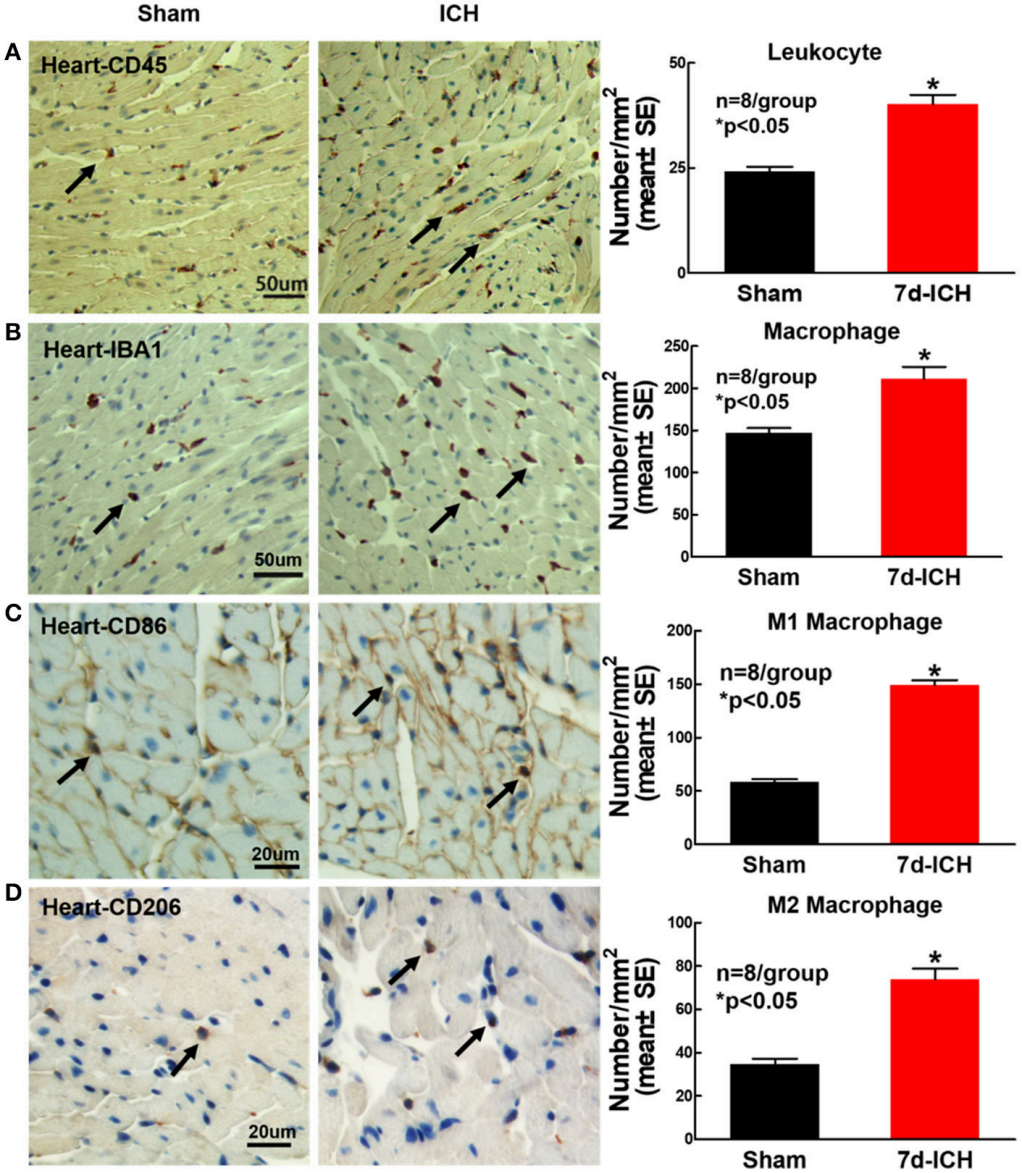
ICH significantly increases cardiac inflammatory cell infiltration compared to sham control mice. ICH increases cardiac inflammatory cells infiltration at 7 days after ICH. **(A)** CD45 (leukocyte marker) and **(B)** IBA1 (macrophage marker) immunostaining and quantitative data at 7 days after ICH. ICH in mice significantly increases **(C)** M1 macrophage indicated by CD86 and **(D)** M2 macrophage indicated by CD206 in heart compared to sham control mice. **(A,B)** scale bar, 50 μm; **(C,D)** scale bar, 20 μm. Data are presented as mean ± SE. ^*^p < 0.05 compared with sham.

To determine whether ICH induces inflammatory cell infiltration into heart at early stage after ICH, leukocyte (CD45) and microphage (IBA1) expression were measured using immunostaining. The phenotype of macrophages was evaluated using M1 marker (CD86) and M2 marker (CD206). The result shows that ICH significantly increases leukocyte (Figure 6A), macrophage (Figure 6B) and M1 (Figure 6C) and M2 (Figure 6D) macrophage infiltration into the heart compared to sham control mice. The data indicate that ICH induces inflammatory cell infiltration into heart compared to sham control mice.

## Discussion

Cardiac complications are common in the ICH patients (29, 30). In this study, we report that ICH in adult mice induces significant and progressive cardiac dysfunction with increased cardiac fibrosis, cardiomyocyte hypertrophy and apoptosis compared to sham control mice. ICH also increases systemic and as well as cardiac inflammatory and oxidative stress compared to sham control mice. We are the first to demonstrate that ICH induces progressive cardiac dysfunction in the absence of primary cardiac disease in mice. Increasing heart inflammation and oxidative stress may contribute to ICH induced heart damage after ICH.

### ICH induces progressive cardiac dysfunction

Acute brain injury may cause cardiac injury (31). Clinical studies indicate that cardiac complications increase in-hospital mortality in ICH patients (64% with increased troponin I compared with 28% with normal troponin I (29, 30). 4.1% of patients suffer at least one acute serious cardiac complication after intracerebral hemorrhage, and acute heart failure was the most common serious in-hospital cardiac event (7). As ICH patients have systemic complications (hypertension, diabetes mellitus, myocardial ischemia, etc.), acute heart failure may occur as a consequence of fluid overload, new-onset myocardial ischemia, or high BP. Whether ICH directly induces cardiac dysfunction remains poorly understood. In this study, we focus on the interaction between ICH and heart injury. Our data indicate that ICH not only induces acute and chronic cardiac dysfunction identified by decreased LVEF and LVFS, but also increases chronic cardiac pathological remodeling identified by increased cardiac interstitial fibrosis and cardiomyocyte hypertrophy compared to sham control mice. Compared with acute (7 days) ICH, the ICH mice at a chronic stage (28 days) exhibited more severe and progressive deficits identified by decreased LVEF, LVFS, IVS, and increased LVID, LV Volume. To our knowledge, our data are the first to demonstrate that ICH induces progressive cardiac deficit in the absence of primary cardiac disease in mice.

### ICH increases inflammatory cell infiltration into heart and increases inflammatory factor expression in heart tissue

Clinical studies found systemic activation of the immune system after ICH which may influence outcome in ICH patients (32). ICAM-1, a transmembrane protein molecule, is found in low concentrations in the membranes of leukocytes, activated lymphocytes and endothelial cells under normal physiological conditions (33). However, it is rapidly up-regulated by cytokine stimulation, enhancing adhesion of leukocytes to endothelial cells (34). When activated, leukocytes bind to endothelial cells via ICAM-1 signaling, and then ICAM-1 promotes leukocyte transmigration into tissues (34, 35). Circulating ICAM-1 levels are significantly increased following ischemic stroke in patients (36). MCP-1 is a small, pro-inflammatory cytokine that recruits inflammatory monocytes into various tissues (37). Clinical studies have found that myocardial infarction and brain ischemic stroke both significantly increase circulating MCP-1 level when compared to health controls (38). ICAM-1 and MCP-1 can be induced by the pro-inflammation molecules interleukin-1 (IL-1) and TNF. Our pre-clinical studies also found that ischemic stroke and TBI in mice significantly increase serum and heart ICAM-1 and MCP-1 expression as well as induce heart deficits (5, 10). In the present study, we found that ICH significantly increases ICAM-1, MCP-1 and IL-1 expression in the heart and serum as well as increases inflammatory cells (leukocyte and macrophage) infiltration into the heart after ICH. The increased MCP-1 and ICAM-1 may play a role in increasing inflammatory cell infiltration into the heart after ICH.

Following onset of ICH, there are large numbers of infiltrating macrophages in the peri-hematoma regions as shown by previous studies (39, 40) as well as our data in the present study. In their study, Min et al. demonstrated a significant increase in M1 macrophage markers such as iNOS and CD86 as well as M2 macrophage markers such as Arginase-1 and Ym1 at 7 days after a collagenase induced ICH model in mice (39). The glial cells of the brain likely facilitate M2 polarization of the infiltrating macrophages to facilitate repair and recovery after ICH (39). In our study, we found a significant increase in M1 (CD 86) as well as M2 (CD206) macrophages in brain as well as heart tissue at 7 days after ICH which is consistent with their findings. In the present study, we focus our investigation on whether inflammatory responses participate in ICH induced heart damage.

Inflammatory cell infiltration into the heart may induce cardiac inflammatory factor expression. The invasion of macrophages not only causes direct damage to the heart, but also contributes to release MCP-1, TGF-β and MMP-9, which thereby exacerbates the cardiac damage (41). Mewhort et al. reported that systemic monocytes increase cardiac myofibroblast activity and release of TGF-β and MMP-9 thereby, inducing local extracellular matrix (ECM) remodeling (42). Peripheral blood monocytes co-cultured with myofibroblasts under direct contact conditions significantly increase TGF-β1 and MMP-9 level in the culture media (42). Monocyte released TGF-β increases cardiac hypertrophy and fibrosis (42). TGF-β is known to stimulate cardiac myofibroblast activation and increases ECM deposition in the infarct by upregulating collagen and fibronectin synthesis as well as by decreasing matrix degradation (43). Increased TGF-β is related with increased collagen synthesis markers and is correlated with increased MMP-9 level in hypertensive disease patients (44). MMP-9 can be secreted by a wide number of inflammatory cells such as neutrophils, macrophages, and fibroblasts. MMP-9 regulates neutrophil migration across the basement membrane (45), and has been associated with cardiac pathological remodeling and fibrosis in cardiovascular disease (46). TGF-β and MMP-9 both not only promote abnormal cardiac collagen deposition and fibrosis, but also mediate cardiac function (44). Increased TGF-β and MMP-9 levels are related with the impairment of LV longitudinal deformation and abnormal LV twisting (assessed by echocardiography and electrocardiogram) in hypertensive patients as well as impaired arterial elastic function (44). We found that ICH significantly increases TGF-β and MMP-9 expression in the heart as well as induces cardiac deficit after ICH compared to sham control mice. The increased heart inflammation and TGF-β and MMP-9 expression may promote ICH induced cardiac functional deficits.

### ICH induced oxidant stress may contribute to cardiac damage

TGF-β also increases the expression level of NOX-2 (47). NADPH oxidase family enzymes (or NOXs) are a major source of ROS and have been implicated in oxidative damage following brain injury such as trauma, and ischemic or hemorrhagic stroke (48). Oxidative stress also contributes to the pathogenesis of heart failure. NOX-2 promotes cardiomyocyte death and plays an important role in cardiac remodeling following myocardial infarction (49). By promoting the transition of fibroblasts to myofibroblasts, NOX 2 also increases cardiac inflammation and induces fibrosis and cardiomyocyte hypertrophy (50). In our study, we found that ICH significantly increased heart and serum NOX-2 level as well as increased cardiomyocyte apoptosis compared to the sham group. Taken together, ICH significantly increases systemic inflammatory status and oxidative stress, which in concert may mediate ICH-induced cardiac deficits.

### Limitations

Catecholamine released in the post-injury period may play a role in mediating cardiac deficits after brain injury (51). Sympathetic response and elevated systemic catecholamine levels have been associated with cardiac dysfunction in patients after stroke (52). Besides sympathetic nerve terminals which can release catecholamine directly into heart, the adrenal medulla can also release catecholamine into bloodstream which can then reach the heart (51, 52). High levels of circulating catecholamines can exacerbate cardiac damage, but high circulating catecholamine levels are not required for pathological cardiac remodeling (52). Therefore, while catecholamine may play a role in cardiac dysfunction after ICH, there may be several other pathways that mediate cardiac dysfunction after brain injury.

In addition, the nervous system and the immune system affect each other. The nervous system communicates with the immune system via sympathoadrenergic pathways. There is evidence suggesting that all human immune cells (including T and B cells, dendritic cells, macrophages, microglia, and neutrophils) can express dopaminergic receptors (53). Previous studies also suggest the occurrence of endogenous catecholamine in immune cells (54), dopamine, noradrenaline, and adrenaline have been identified and measured in several immune cell types (55, 56). Thus, immune response after brain injury may also play a vital role in mediating brain-heart interaction.

In this study, we establish a direct link between the brain and heart and our data indicate that inflammation and oxidative stress may participate brain-heart interaction after ICH. However, we do not exclude the possibility that several other factors may mediate brain-heart interaction after ICH. The effect of sympathetic activation in mediating cardiac dysfunction after ICH are not tested in the study and further studies to find investigate and identify other mediators of cardiac dysfunction after ICH are warranted.

## Conclusions

Our study demonstrates that ICH induces progressive cardiac dysfunction in the absence of primary cardiac disease in mice. Increasing heart inflammation and oxidative stress may play key roles in mediating ICH-induced cardiac dysfunction.

## Author contributions

WL performed experiments, analyzed data, and wrote the manuscript. LL performed experiments, analyzed data, and prepared figures. MC was involved in experimental design and gave final approval of manuscript. PV performed experiments and wrote the manuscript. AZ, ZC, and JL-W performed experiments. TY was involved in experimental design and gave final approval of manuscript. JC was involved in experimental design, wrote the manuscript, analyzed data, and gave final approval of manuscript.

### Conflict of interest statement

The authors declare that the research was conducted in the absence of any commercial or financial relationships that could be construed as a potential conflict of interest.

## Abbreviations

IBA1: Ionized calcium binding adaptor molecule 1
ICAM-1: Intercellular Adhesion Molecule-1
ICF: Interstitial collagen fraction
ICH: Intracerebral hemorrhage
IL-1: Molecular interleukin-1
IL-1β: Interleukin-1beta
IVS: Interventricular septum
LV: Left ventricle
LVEF: Left ventricular ejection fraction
LVFS: Left ventricular fractional shortening
LVID: Left ventricle interior diameter
MCP1: Monocyte chemotactic protein-1
MCSA: Cardiomyocyte cross-sectional areas
MMP-9: Matrix metalloproteinase-9
NOX-2: Nicotinamide adenine dinucleotide phosphate oxidase-2
PSR: Picro Sirius Red
ROS: Reactive oxygen species
TBI: Traumatic brain injury
TGF-β: Transforming growth factor beta
TNF: Tumor necrosis factor
TUNEL: TdT-mediated Biotin-dUTP Nick End labeling
BP: Blood pressure
DAP: Diastolic arterial pressure
MAP: Mean arterial pressure
SAP: Systolic arterial pressure
H&E: Hematoxylin and Eosin.
